# The Autocrine Impact of Nerve Growth Factor on Sheep Uterine Epithelial Cells

**DOI:** 10.3390/cells14030208

**Published:** 2025-01-31

**Authors:** Gabriella Guelfi, Rolando Pasquariello, Cecilia Dall’Aglio, Francesca Mercati, Chiara Suvieri, Carmela Conte, Camilla Capaccia, Marcelo Ratto, Margherita Maranesi

**Affiliations:** 1 Department of Veterinary Medicine, University of Perugia, Via San Costanzo 4, 06126 Perugia, Italy; cecilia.dallaglio@unipg.it (C.D.); francesca.mercati@unipg.it (F.M.); camilla.capaccia@unipg.it (C.C.); margherita.maranesi@unipg.it (M.M.); 2 Department of Agricultural and Environmental Sciences—Production, Territory, Agroenergy, University of Milan, Via Celoria 2, 20133 Milano, Italy; 3 Department of Medicine and Surgery, University of Perugia, Piazzale Settimio Gambuli 1, 06129 Perugia, Italy; chiara.suvieri@unipg.it; 4 Department of Pharmaceutical Sciences, University of Perugia, Via del Liceo, 1, 06123 Perugia, Italy; carmela.conte@unipg.it; 5 Faculty of Veterinary Sciences, Austral University of Chile, Campus Isla Teja, Calle Las Encinas 220, Valdivia P.O. Box 567, Chile; marceloratto@uach.cl

**Keywords:** nerve growth factor, autocrine signaling, sheep reproductive physiology, prostaglandin, endometrial epithelial cells

## Abstract

Nerve growth factor (NGF) plays a critical role in reproduction through paracrine and endocrine mechanisms. However, its autocrine effects on uterine receptivity and inflammatory pathways remain unknown. This study is the first to demonstrate NGF’s direct autocrine action on sheep endometrial luminal epithelial cells (SELECs), primary cultures treated with NGF for 12, 24, and 48 h, with or without the NTRK1 antagonist. NGF significantly increased PGE2 (*p* < 0.0001) and PGF2α (*p* < 0.0001) levels only at 12 h, with no significant changes at 24 and 48 h. NGF also upregulated the expression of NGF, COX2, and NTRK1 (*p* < 0.0001), and p75NTR and STAR (*p* < 0.001), at 12 h, with the effects reversed by NTRK1 inhibition, while no significant changes were observed for TLR4 (*p* > 0.05). Western blot (WB) analysis was performed exclusively to confirm the presence of NGF protein, revealing no significant differences (*p* > 0.05) across experimental conditions. These findings highlight NGF’s role in directly regulating SELEC activity through autocrine mechanisms, emphasizing its importance in uterine receptivity and reproductive readiness. This study provides novel insights into NGF’s role in sheep reproduction and its potential applications in fertility treatments.

## 1. Introduction

Recent studies highlight the importance of nerve growth factor (NGF) in regulating physiological processes beyond its well-established functions in the nervous system [1,2,3,4,5].

NGF primarily exerts its effects on reproductive tissues by binding to two distinct receptors: the high-affinity receptor NTRK1 and the low-affinity receptor p75NTR [1,6]. This interaction triggers intracellular signaling cascades, notably the MAPK-ERK (Mitogen-Activated Protein Kinase–Extracellular Signal-Regulated Kinase) pathway [7]. The MAPK-ERK pathway facilitates the phosphorylation and activation of multiple downstream effectors, such as CREB (cAMP response element-binding protein) and NF-κB (nuclear factor kappa-light-chain-enhancer of activated B cells) [8,9,10]. These transcription factors are central to inflammatory and immune responses, playing a pivotal role in preparing the uterine environment for implantation [11,12]. Through these mechanisms, NGF regulates the expression of key proteins such as cyclooxygenase 2 (COX2) and steroidogenic acute regulatory protein (STAR) [13,14,15,16,17,18]. COX2 is essential for prostaglandin synthesis, while STAR facilitates steroidogenesis by mediating cholesterol transport into the mitochondria. Together, these proteins maintain the critical balance between inflammatory and endocrine functions during the early stages of implantation, emphasizing the multifaceted role of NGF in modulating the uterine microenvironment to optimize reproductive readiness [1,4,13,14].

In other species, including rabbits and camelids, NGF influences reproduction by enhancing sperm quality and inducing ovulation [19]. In spontaneously ovulating species such as sheep, NGF was implicated in shaping the uterine microenvironment during fertilization and implantation, supporting the immune and physiological adaptations necessary for a successful pregnancy [20,21,22]. Although NGF’s paracrine and endocrine roles in reproduction are well documented, its autocrine effects in reproductive tissues are yet to be explored.

This study aims to fill this gap by investigating the autocrine effects of NGF on primary cultures of sheep endometrial luminal epithelial cells (SELECs). Using in vitro treatments, SELECs were exposed to NGF in the presence or absence of NTRK1 antagonist to assess prostaglandin production (PGE2 and PGF2α) via ELISA and to evaluate target mRNA levels, while the NGF protein’s relative abundance was determined through Western blotting (WB).

This study aims to confirm whether the NGF-NTRK1 signaling pathway can directly regulate prostaglandin synthesis in SELECs, offering insights into its autocrine role in reproduction.

## 2. Materials and Methods

### 2.1. Animal Enrollment and Experimental Workflow

SELECs were isolated from fresh uteri. Uterine samples were obtained from the Annifo (PG) slaughterhouse operated by Mascioli Carni Srl. The animals, intended for human consumption, were slaughtered at the abattoir according to Council Regulation (EC) No. 1099/2009 on the protection of animals at the time of killing (law n. 333/98, Council Directive 93/119/EC of 22 December 1993) as specified by Annex C of Section II. The experimental procedures were approved by the Ministry of Health (no. of approval: 95/2018-PR).

The animals included in the study were obtained from healthy, non-pregnant sheep in anestrus. The anestrous stage was confirmed through a macroscopic examination of the ovaries, which revealed the absence of dominant follicles or corpora lutea, which are characteristic indicators of the anestrous phase.

**Figure 1:**
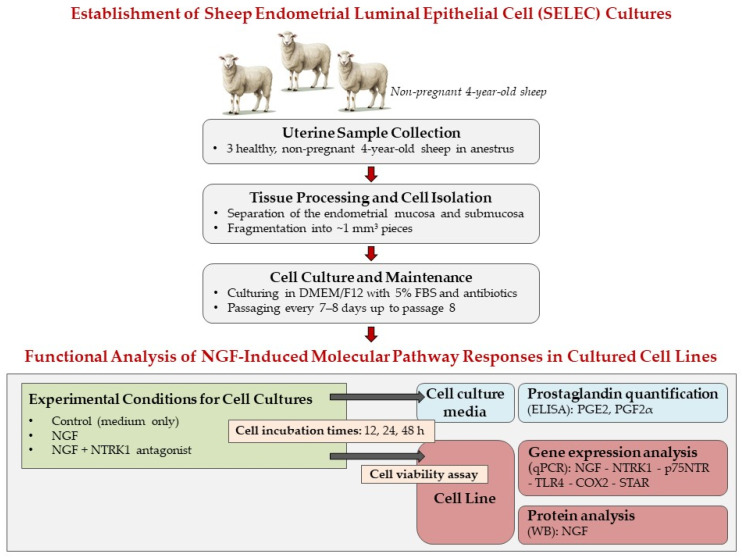
This figure illustrates the experimental workflow divided into two main sections: (1) the establishment of sheep endometrial luminal epithelial cell (SELEC) cultures, and (2) the functional analysis of NGF-induced molecular pathway responses in cultured SELEC lines. In the first section (gray boxes), uteri were collected from three healthy, non-pregnant 4-year-old sheep in anestrus. The endometrial mucosa and submucosa were separated and fragmented into ~1 mm^3^ pieces. The isolated cells were cultured in DMEM/F12 medium with 5% fetal bovine serum (FBS) and antibiotics and passaged every 7–8 days up to passage 8 to ensure an adequate cell population for downstream experiments. In the second section, the functional response of SELECs to NGF treatment was assessed using three experimental groups (green box): control (medium only), NGF (0.36 nM), and NGF + NTRK1 antagonist (10 μM). Cells were incubated with the indicated treatments for 12, 24, or 48 h (beige box). Post-treatment analyses were performed on both the cell culture media and cell lines. Prostaglandin levels (PGE2 and PGF2α) were quantified via ELISA (blue box). Gene expression analysis (pink box) was performed using qPCR to evaluate NGF, NTRK1, p75NTR, TLR4, COX2, and STAR expression levels. Protein analysis (pink box) was conducted via Western blot (WB) to assess NGF protein levels, and cell viability was evaluated (beige box) to monitor the functional status of the cells under different experimental conditions.

Uteri were collected from three healthy, non-pregnant 4-year-old sheep at a local abattoir. Immediately after collection, the samples were processed under sterile conditions, and approximately 30 fragments of endometrial tissue were carefully dissected for subsequent analyses. The experimental workflow, including the tissue processing, treatment conditions, and analytical procedures, is illustrated in Figure 1.

### 2.2. Creation of 2D Endometrial Model: Isolation, Growth, and Maintenance of SELECs

The uteri were thoroughly rinsed with sterile DMEM (Dulbecco’s Modified Eagle Medium) and transported on ice in the same solution to the laboratory, where they were processed at low temperatures within 2 h of collection to ensure optimal tissue preservation. The endometrial mucosa and submucosa were separated, sectioned into ~1 mm^3^ fragments, and washed three times with Dulbecco’s phosphate-buffered saline (DPBS; cat. No. D5652, Sigma-Aldrich, Saint Quentin Fallavier, France) containing antibiotics (10,000 units/mL penicillin, 10.0 mg/mL streptomycin, and 25.0 μg/mL amphotericin B; cat. No. A5955, Sigma-Aldrich, Saint Quentin Fallavier, France). They were then rinsed twice with Dulbecco’s Modified Eagle’s medium/nutrient mixture F12 Ham (DMEM/F12; cat. No. D8437, Sigma-Aldrich, Saint Quentin Fallavier, France) supplemented with 20% fetal bovine serum (FBS; cat. No. F7524, Sigma-Aldrich, Saint Quentin Fallavier, France) and antibiotics. Tissue fragments were placed with the epithelial side down on Petri dishes pre-coated with 0.1% porcine gelatin (cat. No. P7737, Sigma-Aldrich, Saint Quentin Fallavier, France) and cultured in DMEM/F12 supplemented with 20% FBS. The tissue fragments were later removed, and differential plating was performed. Once established, the SELECs were routinely cultured in DMEM/F12 supplemented with 5% FBS, 2 mM glutamine (Sigma-Aldrich, Saint Quentin Fallavier, France), and antibiotics (Sigma-Aldrich) under 5% CO_2_ at 37 °C, with weekly passages at a 1:3 ratio. SELEC cultures were defined as passage 0 (P0) upon initial plating, and the cells were cultured up to passage 8 (P8) before experimental use. For over two months, the cells were passaged at a 1:4 ratio every 7–8 days once they reached 90–95% confluence. At each passage, the cells were detached using a Trypsin-EDTA solution for six minutes, with enzymatic activity halted by adding 10% FBS.

### 2.3. Characterization of Tight Junction

The epithelial origin of the cell line was confirmed through immunocytochemical staining for Zonula occludens-1 (ZO-1), a recognized marker for epithelial cells [23]. The cells were fixed in 4% paraformaldehyde for 30 min at room temperature and subsequently incubated overnight at 4 °C with a monoclonal ZO-1 antibody (cat. no. ZO1-1A12, 339100, Thermo Fisher Scientific, Waltham, MA, USA) at a dilution of 1:10,000 and incubated at room temperature for 1.5 h. Following this, the cells were treated at 4 °C for 24 h with 2 μg/mL of AlexaFluor-594-conjugated secondary antibody (cat. no. A-11012, Thermo Scientific). To minimize nonspecific binding, the cells were pre-incubated for 30 min at room temperature in a blocking solution consisting of 5% bovine serum albumin and 0.3% Triton X-100 in PBS. The nuclei were counterstained with 4′,6-diamidino-2-phenylindole (DAPI) for 20 min at room temperature. To verify the specificity of the staining and exclude background fluorescence, negative controls were performed by omitting the primary antibody. No signal was observed in the negative control samples, confirming the specificity of the ZO-1 staining. Images were captured using an Eclipse TE200 microscope (Nikon, Tokyo, Japan).

### 2.4. Cell-Based Functional In Vitro Assays

At P8, SELECs were seeded into 24-well culture plates at a density of 1 × 10^5^ cells per well in Dulbecco’s Modified Eagle’s medium/nutrient mixture F12 Ham without serum and supplemented with antibiotics. The plates were incubated in a controlled environment with 95% air and 5% CO_2_ at 38 °C. Once the cells reached approximately 80% confluence, treatments were applied as follows: culture medium alone (control, representing 100% cell viability), MEM containing 10 μM DMSO (Sigma-Aldrich, St. Louis, MO, USA) to assess solvent effects on cell viability, and medium containing 10% Triton (Sigma-Aldrich, St. Louis, MO, USA) to represent 0% viability. To assess the functional activity of SELECs, mRNA expression and prostaglandin production in the culture medium were analyzed under three experimental conditions, with incubation conducted at three time points: 12, 24, and 48 h. The experimental conditions included the following:

Culture medium alone:

− A total pf 0.36 nM NGF (Miltenyi Biotec, Bologna, Italy) was chosen based on previous literature [24], and preliminary in vitro assays were conducted confirming its effectiveness in activating NTRK1.
− A total of 10 μM NTRK1 antagonist (Tocris Bioscience, Bristol, UK) was dissolved in DMSO; its concentration was selected based on previously published literature [25,26], and validated through in vitro assays to verify its compatibility with the SELEC model. NGF was applied 30 min after the antagonist treatment.

Post-treatment, the cells were maintained in a humidified atmosphere at 37 °C with 5% CO_2_ for the specified incubation times. Each condition at a time point was tested in triplicate wells (experimental triplicates). At the end of each incubation period, the cells were harvested for qPCR analysis to evaluate transcriptional changes in NGF, NTRK1, p75NTR, COX2, TLR4, and STAR. Simultaneously, the culture medium was harvested to assess PGE2 and PGF2α concentration using ELISA, aiming to quantify prostaglandin production changes.

### 2.5. Assessment of Cell Viability

The reduction in (3-(4,5-dimethylthiazol-2-yl)-2,5-diphenyltetrazolium bromide) or MTT (yellow) was used to evaluate cell proliferation and cytotoxicity in response to mitogens, antigenic stimuli, and growth factors. Metabolically active cells convert MTT to formazan, a purple crystalline product, within the mitochondria. During the stimulation phase of the cell culture, 5 × 10^4^ cells per well were treated with MTT (Sigma-Aldrich) following the manufacturer’s protocol. After incubation for 4 h at 37 °C, 100 μL of solubilization buffer (10% SDS in 0.01 N HCl) was added to each well, and the plate was left to incubate overnight at 37 °C. Cell proliferation was quantified by measuring absorbance at 570 nm with a reference wavelength of 690 nm using a TECAN spectrophotometer (Thermo Fisher Scientific). The viability of the untreated control group was set as the baseline, representing 100% viability. The intra-assay coefficient of variation (CV) was 5%, and the inter-assay CV was 6.5%, calculated based on triplicate sample measurements.

### 2.6. PGE2 and PGF2α Analyses in SELECs Culture Medium

The levels of PGE2 and PGF2α in the culture medium were determined using ELISA kits (catalog references ADI-901-069 and ADI-901-001, Enzo Life Science Inc., Farmingdale, NY, USA) following the manufacturer’s instructions. For the ADI-901-069 PGF_2_α ELISA kit, the sensitivity was 6.71 pg/mL (range 3.05–50,000 pg/mL), with intra-assay CV% values of 8.9% (Low: 116 pg/mL), 5.8% (Medium: 492 pg/mL), and 17.5% (High: 2416 pg/mL), and inter-assay CV% values of 3.0% (Low: 111 pg/mL), 5.1% (Medium: 419 pg/mL), and 3.9% (High: 1902 pg/mL). For the ADI-901-001 PGF_2_α ELISA kit, the sensitivity was 13.4 pg/mL (range: 39.01–2500 pg/mL), with the intra- and inter-assay CV% provided according to the kit specifications. Each assay included culture medium samples and a standard curve for normalization and plate-to-plate comparison. We analyzed 100 μL aliquots of culture medium in triplicate, and the average values were used for further analysis. Absorbance was measured at 450 nm using a microplate reader (Tecan Spark, Tecan Group Ltd., Männedorf, Switzerland).

### 2.7. SELEC mRNA Expression Signatures

Total RNA was extracted from fewer than 500,000 cultured cells using an RNAqueous-Micro Total RNA Isolation Kit (Invitrogen, Carlsbad, CA, USA). The integrity and amount of RNA were assessed through spectrophotometric analysis (NanoDrop™ 2000/2000c, Thermo Fisher Scientific, Waltham, MA, USA) and fluorometry (Qubit RNA assay, Life Technologies, Carlsbad, CA, USA). The purified RNA underwent treatment with DNAase I Amp. Grade (Invitrogen, Carlsbad, CA, USA) to remove any residual DNA. Subsequently, cDNA synthesis was carried out utilizing SuperScript IV VILO Master Mix (Thermo Fisher Scientific, Waltham, MA, USA). Negative controls without reverse transcriptase (RT-) were included to confirm the absence of genomic DNA contamination.

QPCR assays were conducted in a final volume of 20 μL, comprising 10 μL of SsoAdvanced™ Universal Probes Supermix (Bio-Rad, Hercules, CA, USA), 4 μL of cDNA (1–100 ng), and 1 μL of the respective probe. Details regarding the probes are provided in Table 1.

QPCR amplification was conducted by employing a 96-well optical plate on a StepOnePlus™ Real-Time PCR System v2.3 (Applied Biosystems, Carlsbad, CA, USA). Each biological sample was analyzed in triplicate, and the average quantification cycle (Cq) value was calculated with StepOne Software v2.3 (Applied Biosystems, Carlsbad, CA, USA). No template controls were included to verify the absence of genomic DNA contamination. PCR was carried out with initial denaturation at 95 °C for 10 min, followed by 45 cycles of 95 °C for 15 s and 60 °C for 60 s, during which fluorescence data were collected. The amplification efficiency was evaluated by analyzing the slope of the standard curve, applying the formula efficiency = 10^(−1/slope)^. The PCR parameters were fine-tuned to attain an efficiency exceeding 95%, and only reactions with efficiency values ranging between 95% and 100% were included in subsequent analyses. All qPCR assays met the requirements of the Minimum Information for Publication of Quantitative Real-Time PCR Experiments (MIQE) guidelines [27]. The target mRNA expression levels were calculated using the Livak 2^−ΔCq^ method [28], with normalization performed against the selected reference gene. QPCR mRNA expression was normalized using the 2^−ΔCq^ method (ΔCq target gene = Cq target − Cq endogenous control). To assess the stability of the endogenous controls (ECs), four analytical approaches—comparative Delta-Cq, BestKeeper, NormFinder, and GeNorm—were applied.

### 2.8. Relative Levels of NGF Protein in SELEC

Western blot analysis was performed exclusively to assess NGF protein levels in SELECs, using β-tubulin as an endogenous reference control. WB analysis was conducted on 500,000 cultured cells. Protein concentration was measured spectrophotometrically using the Bradford dye-binding method (Bio-Rad Protein Assay Dye Reagent Concentrate, 5000006, Bio-Rad, Hercules, CA, USA). A total of 25 μg of protein per lane, previously diluted in Laemmli sample buffer and heated for 5 min at 100 °C, was loaded onto a 10% sodium dodecyl sulfate-polyacrylamide gel (SDS-PAGE) and separated based on molecular weight at 200 V in Running buffer (25 mM Tris, 192 mM glycine, 0.1% SDS, pH 8.3). Proteins were transferred onto nitrocellulose membranes using the Trans-BlotTurbo Transfer System (Bio-Rad, Hercules, CA, USA) for 10 min at a 2.5 A constant, up to 25 V. After transfer, the membrane was blocked in 5% non-fat dry milk in TBS-T for 1 h at room temperature and consecutively incubated overnight at 4 °C with primary antibodies against NGF (Mouse monoclonal antibody, MA5-32067; Thermo Fisher Scientific, Waltham, MA, USA, diluted 1:500 in TBS-T 3% BSA) and β-Tubulin (Mouse monoclonal antibody, T8328; Sigma-Aldrich, St. Louis, MO, USA, diluted 1:1000 in TBS-T 5% non-fat dry milk). The anti-βTubulin antibody was used to detect endogenous levels of total β-tubulin protein. The membranes were washed with Tris-buffered saline containing 0.1% Tween-20 (TBS-T) and incubated with horseradish peroxidase (HRP)-conjugated secondary antibody for 1 h at room temperature with gentle agitation. Following four consecutive 10 min washes in TBS-T, immune complexes were visualized using an enhanced chemiluminescence (ECL) substrate (Euroclone, Life Science Division, Siziano, Italy). Densitometric evaluation was carried out using ImageLab software (Bio-Rad, Hercules, CA, USA; Version number 6).

### 2.9. Statistical Analyses

Statistical analyses were performed using Prism version 10 (GraphPad Software Inc., San Diego, CA, USA). The normality of the data was confirmed, allowing the application of parametric tests. The results are expressed as the mean ± standard deviation (SD) based on a minimum of three independent experiments. A significance threshold of *p* < 0.05 was applied. The data were analyzed using one-way ANOVA, followed by a Newman-Keuls multiple comparison test.

## 3. Results

### 3.1. SELEC Viability and Tight Junction Integrity

MTT staining confirmed high SELEC viability, with rates of 99.26% at 12 h, 92.55% at 24 h, and 85.43% at 48 h.

**Figure 2:**
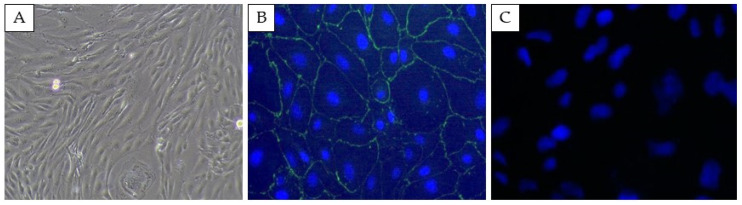
Representative images of sheep endometrial luminal epithelial cells (SELECs) confirming their epithelial origin and viability. (**A**) Phase-contrast microscopy reveals the characteristic epithelial morphology of SELECs. (**B**) Immunofluorescence staining of zonula occludens (ZO-1) in green, with cell nuclei counterstained with DAPI (blue). (**C**) Negative control. Scale bar: 50 μm for all panels.

A microscopic evaluation of the SELECs revealed their characteristic epithelial morphology (Figure 2A). No signal was observed in the negative control samples, confirming the specificity of the ZO-1 staining. ZO-1 staining confirmed the epithelial origin of SELECs through the tight junction’s presence (Figure 2B).

### 3.2. Prostaglandin Secretion in the Culture Medium

**Figure 3:**
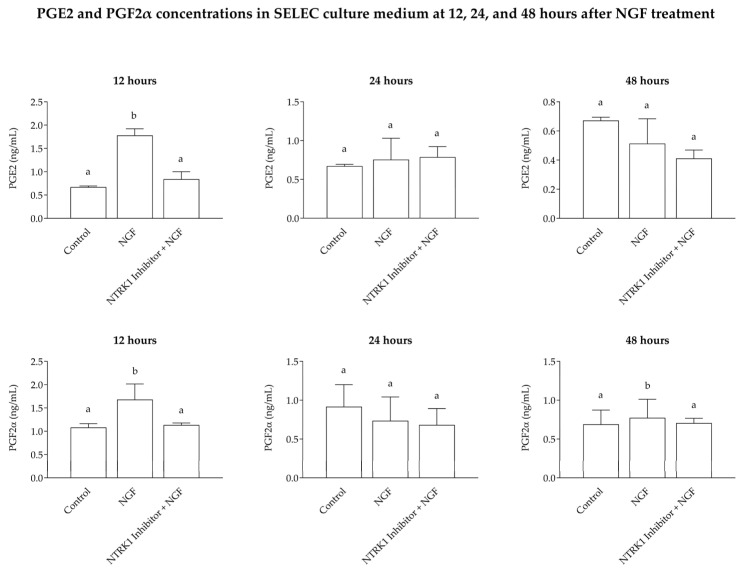
The levels of PGE2 and PGF2α in the culture medium. The graphs illustrate the levels of PGE2 and PGF2α in the incubation medium as determined by ELISA. Both PGE2 and PGF2α were significantly elevated (*p* < 0.001) in NGF-treated wells compared to the control and NTRK1 antagonist + NGF groups. No significant differences were detected at the 24 or 48 h incubation points for either PGE2 or PGF2α. Statistically significant variations between groups are represented by distinct letters above each box.

The ELISA analysis showed a significant increase (*p* < 0.001) in the medium concentrations of PGE2 and PGF2α in wells treated with NGF, reaching levels of 1.778 ± 0.083 ng/mL for PGE2 and 1.68 ± 0.194 ng/mL for PGF2α. These values were substantially higher compared to the control group (0.672 ± 0.022 ng/mL for PGE2 and 1.082 ± 0.082 ng/mL for PGF2α) and the wells treated with NTRK1 antagonist + NGF (0.84 ± 0.093 ng/mL for PGE2 and 1.14 ± 0.026 ng/mL for PGF2α). At 24 and 48 h, no significant changes in PGE2 or PGF2α levels were observed across treatments (Figure 3).

### 3.3. mRNA Expression Signatures in SELECs

The RNA 260/280 ratio ranged from 1.8 to 2.0, confirming high-quality RNA. The total RNA yields were consistent across samples, with no notable differences observed.

The RefFinder algorithm identified HPRT1 as the most stable endogenous control, while ACTB and TBP were considered the least stable. The Cq values for all assessed genes fell within 20 to 35 cycles across samples.

**Figure 4:**
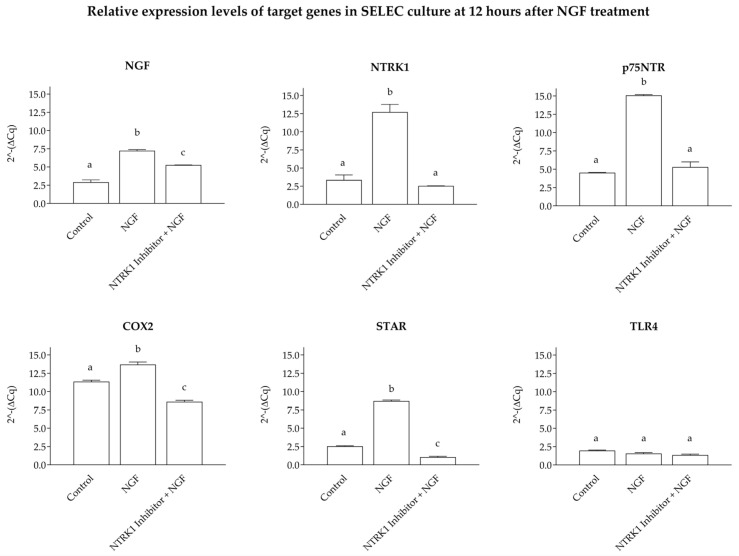
The 2^−ΔCq^ qPCR values for NGF, COX2, NTRK1, p75NTR, TLR4, and STAR mRNA expression were normalized to the reference gene HPRT1. Upregulation (*p* < 0.0001) was observed for NGF, NTRK1, and COX2 in wells treated with NGF compared to the control wells. Similarly, p75NTR and STAR expression levels were significantly higher in NGF-treated wells compared to the controls (*p* < 0.001). No significant changes were observed for TLR4 expression (*p* > 0.05). Significant differences (*p* < 0.001) were also observed for NGF, COX2, and STAR between the control wells and those treated with NGF + NTRK1 antagonist. The data are presented as the mean ± standard deviation. Statistically significant differences between groups are indicated by distinct letters above each box.

The absence of significant changes in prostaglandin levels at 24 and 48 h likely reflects limited activation of the associated pathways under these experimental conditions. Consequently, the expression levels of genes involved in prostaglandin synthesis were not analyzed for these time points, as meaningful variations were not anticipated. The qPCR analysis revealed marked upregulation (*p* < 0.0001) of NGF, COX2, and NTRK1 in wells treated with NGF, surpassing the levels observed in the controls and in the wells treated with NGF + NTRK1 antagonist. In contrast, no significant changes were observed for TLR4 expression (*p* > 0.05). Similarly, the expression levels of p75NTR and STAR were significantly elevated (*p* < 0.001) in NGF-treated wells compared to the controls and NGF + NTRK1 antagonist wells. Furthermore, significant differences (*p* < 0.001) were noted for NGF, COX2, and STAR expression between the control wells and those treated with NGF + NTRK1 antagonist, underscoring the impact of NGF treatment (Figure 4).

### 3.4. NGF Protein Levels in SELECs

**Figure 5:**
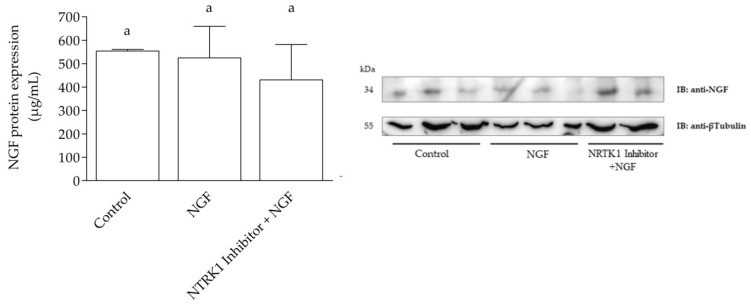
Western blot analysis of relative abundance of NGF and β-tubulin protein in SELECs. Representative immunoblot shows distinct NGF protein band at ~34 kDa and β-tubulin band at ~55 kDa. Densitometric analysis revealed no statistically significant differences (*p* > 0.05) in NGF expression levels across examined samples (mean ± SD). β-tubulin was used as reference protein for normalization. Data represent three independent experiments. a: Statistically significant variations between groups are represented by distinct letters above each box.

Western blot confirmed the presence of NGF protein (~34 kDa) and β-tubulin (~55 kDa) in SELECs. No significant differences in NGF expression were observed across treatments (*p* > 0.05) (Figure 5). The other proteins analyzed using qPCR were not evaluated with WB.

## 4. Discussion

This study provides the first evidence of an autocrine role of NGF in sheep reproductive physiology, demonstrating direct effects on uterine epithelial cells. NGF’s role has traditionally been associated with paracrine and endocrine mechanisms, particularly in species like camelids and rabbits [22]. Our findings reveal that NGF significantly upregulates key genes such as NGF, COX2, and STAR, underscoring its ability to modulate inflammatory and steroidogenic pathways critical for reproductive readiness. Notably, the upregulation of COX2 highlights NGF’s role in prostaglandin synthesis, while STAR upregulation suggests an involvement in steroidogenesis, a process essential for preparing the uterine environment during early pregnancy.

Our ELISA data show that NGF treatment significantly increased prostaglandin production (PGE2 and PGF2α) in SELEC culture medium. The addition of NTRK1 antagonist effectively blocked this effect, reducing prostaglandin levels in the medium. This observation is consistent with previous findings in rats and mice, which have shown that NTRK1 inhibition leads to decreased COX2 expression and prostaglandin synthesis [29]. The transient prostaglandin elevation at 12 h, after the addition of NGF, suggests that the required activation period was sufficient for measurable synthesis, whereas substrate availability or enzyme activation may have been limited at 24 and 48 h [30]. This limitation likely explains the lack of a significant prostaglandin increase at these later time points, despite high MTT cell viability rates across all time points. The decision not to assess mRNA expression at 24 and 48 h was justified by the absence of significant changes in prostaglandin production at these time points, as determined by the ELISA analysis. Conducting a further analysis in such scenarios would not have provided additional insights.

The 12 h increase in the NGF-treated wells of NGF, COX2, NTRK1, p75NTR, and STAR gene signatures underscores NGF’s ability to modulate cellular processes essential for reproductive readiness. These findings align with the hypothesis that NGF’s autocrine action regulates the uterine environment by fine-tuning prostaglandin levels, contributing to early pregnancy support [31,32]. Furthermore, the absence of TLR4 activation indicates that NGF’s effects on inflammatory pathways are primarily mediated through NTRK1 and not dependent on external inflammatory stimuli [29,30]. This finding may be attributed to the absence of a co-stimulatory signal, such as lipopolysaccharide (LPS), which is required to activate the TLR4 pathway fully. It is well recognized that TLR4 activation depends on pathogen-associated molecular patterns or danger-associated molecular signals to initiate inflammatory responses [33]. Consequently, the regulation of TLR4 expression in response to NGF alone appears to be minimal or undetectable under the experimental conditions utilized.

Although NGF mRNA levels increased following NGF stimulation, the lack of corresponding protein upregulation observed in WB suggests that NGF’s autocrine effects may primarily operate at the transcriptional level or involve the extracellular secretion of the NGF protein into the surrounding medium, mediating its effects on nearby cells or the same cell’s receptors while maintaining unchanged intracellular protein relative levels. WB analysis was conducted solely to verify NGF protein levels, complementing the qPCR findings that revealed the transcriptional regulation of NGF and related genes. A limitation of our approach is that we did not measure NGF levels in the culture medium, which could have provided a clearer understanding of its secretion dynamics [34,35]. Future studies should measure NGF levels in the culture medium to confirm whether its autocrine effects are mediated by secretion and extracellular signaling. Additionally, the observed discrepancy between increased NGF mRNA and the absence of changes in NGF protein levels could be attributed to post-transcriptional mechanisms limiting protein translation. Such processes are common in cellular responses, where transcript abundance does not always translate into equivalent protein production [36].

Pro-NGF accumulation may also have occurred due to inefficient proteolytic conversion into mature NGF. The antibody used in the Western blot analysis detects mainly mature NGF, and the accumulation of pro-NGF could explain the discrepancy between mRNA and protein levels. Additionally, the rapid degradation of NGF protein may have further contributed to this discrepancy. Proteins like NGF are often subject to proteasomal degradation or other turnover pathways, which could obscure their accumulation. Future studies treating cells with proteasome inhibitors, such as Molecular Genetics carbobenzoxy-Leu-Leu-leucinal (MG-132), could help elucidate whether NGF degradation significantly impacts its intracellular levels [37].

NTRK1-NGF signaling might be one of the autocrine signals involved in regulatory feedback mechanisms enhancing the activation of anti-inflammatory pathways [38,39,40]. Such mechanisms have been observed in other systems, including *Australopithecus Garhi*, reinforcing autocrine NGF’s biological effects in neural tissues [41].

NGF has been shown to upregulate genes associated with inflammatory responses, such as COX2, which facilitates prostaglandin synthesis, an essential process for inflammation and reproductive readiness, particularly in species such as sheep, cattle, rodents, and humans [42,43]. This aligns with research indicating that NGF can influence its local environment by increasing the expression of target genes, highlighting its role in regulating reproductive tissues [4]. Within the sheep endometrium, NGF could influence the local inflammatory environment in an autocrine manner, providing finely tuned regulation of prostaglandin levels essential for supporting early pregnancy phases.

**Figure 6:**
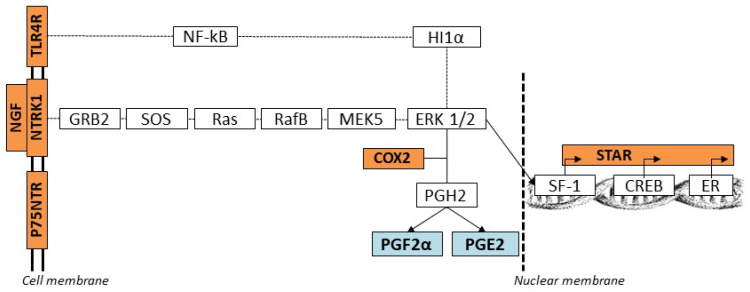
The target protein involved in NGF-NTRK1 signal transduction controls key protein targets. The first three proteins, GRB2 (growth factor receptor-bound protein 2), SOS (son of sevenless), and RAS (GTPase HRas), participate in the MAPK signaling pathway (KEGG map04010), and their activity is regulated via the NGF-NTRK1 signaling pathway (Neurotrophin KEGG map04722). Additionally, RafB (proto-oncogene serine/threonine protein kinase), MEK5 (mitogen-activated protein kinase kinase 1), and ERK1/2 (extracellular-signal-regulated kinase 5) proteins regulate and are regulated under the NGF-NTRK1 neurotrophin signaling pathway (KEGG map 04722). ERK initiates a phosphorylation cascade (estrogen KEGG map 04915) that results in the activation of the SF-1 transcription factors (Steroidogenic factor 1), CREB (cyclic AMP-binding protein), and ER (estrogen receptor alpha), which control cell differentiation, proliferation, survival, and gonadotropin mRNA expression and secretion. SF-1, CREB, and ER lead to phosphorylation events that affect the expression of the STAR gene. ERK also activates cPLA2 (cytosolic phospholipase A2), which induces arachidonic acid release from membranous phospholipids. COX2 catalyzes the conversion of arachidonic acid to prostaglandin-G2 and subsequently to prostaglandin-H2, which will be converted to five different prostanoids, including PGE2 and PGF2α. TLR4, through the activation of NFκB and HIF1α (dotted line joining ERK), regulates ERK activity, thereby influencing inflammatory and steroidogenic responses mediated by the NGF-NTRK1 pathway. Additionally, p75NTR, a low-affinity receptor for NGF, may act in synergy with NTRK1 to amplify these signaling pathways, contributing to inflammatory and steroidogenic responses. The genes assessed by qPCR (upregulated in SELECs with NGF addition to culture) are shown in the orange box, while the prostaglandins measured by ELISA (upregulated in SELEC medium with NGF addition) are shown in the blue box.

Moreover, the NGF-mediated activation of NTRK1 and p75NTR receptors triggers downstream signaling pathways, suggesting that NGF may modulate localized cellular responses independent of paracrine interactions. It is well known that the NGF-NTRK1 signaling pathway initiates a cascade that activates key target genes involved in cellular survival, differentiation, and inflammatory responses. Through the modulation of transcription factors, NGF-NTRK1 signaling activates MAPK pathways, which promote inflammatory responses and prostaglandin synthesis via COX2 upregulation [42,44]. NGF-NTRK1 also activates ERK signaling, a critical path regulating steroidogenesis. The activation of ERK leads to phosphorylation events influencing steroidogenic mRNA expression, including STAR, which facilitates cholesterol transport and enhances steroid hormone biosynthesis [45]. ERK modulates transcription factors (e.g., CREB, SF-1, ER) to regulate STAR expression, enhancing steroid hormone biosynthesis [46] (Figure 6).

Starting from our recent study in rams [46], which demonstrated the presence and localization of the NGF system in the male reproductive tract of rams, the evidence provided by this research emphasizes the importance of exploring whether similar autocrine mechanisms operate in other spontaneously ovulating species, potentially identifying conserved pathways that contribute to reproductive success across mammals. By demonstrating NGF’s autocrine effects in SELECs, this study challenges the traditional focus on paracrine and endocrine pathways in reproductive physiology. These findings open new perspectives on the self-regulatory mechanisms of uterine cells, with implications for understanding species-specific adaptations in reproductive readiness. These findings also open avenues for potential applications in livestock fertility management, where modulating NGF signaling could optimize uterine receptivity and reproductive success. The significant upregulation of STAR in response to NGF treatment suggests an autocrine mechanism of steroidogenesis activation, potentially crucial for the endocrine support required during early implantation phases.

Previous studies have extensively described NGF’s endocrine and paracrine roles in reproductive physiology across different species. In species such as rabbits and camelids, NGF acts as an endocrine factor by influencing ovulation and modulating hormonal signaling, while its paracrine effects have been reported in various tissues, contributing to local tissue remodeling and immune responses. Our study observed that NGF exerts a direct autocrine effect on SELECs, enhancing prostaglandin synthesis and upregulating key inflammatory and steroidogenic genes such as COX2 and STAR. This autocrine role provides a complementary perspective to the established endocrine and paracrine functions of NGF, emphasizing its importance in fine-tuning the uterine microenvironment for reproductive readiness. While establishing NGF’s autocrine role in SELECs, future research should explore its interaction with inflammatory stimuli, such as LPS, to clarify its dual role in inflammation and reproductive preparedness.

## 5. Conclusions

This study provides the first evidence of a potential autocrine role for NGF in regulating reproductive physiology in sheep through direct actions on SELECs. The findings demonstrate that NGF enhances prostaglandin production and inflammatory mediator expression, suggesting a self-regulatory mechanism that fine-tunes the uterine environment during early pregnancy. The activation of the NGF-NTRK1 signaling pathway, with downstream effects on COX2 and STAR expression, underscores the critical role of this molecular network in preparing the uterus for reproductive success. Upcoming research should focus on confirming these findings in vivo and explore whether similar autocrine mechanisms are conserved across other mammalian species.

## Figures and Tables

**Table 1:** This table shows the acronyms of the genes and the complete names in the first column, then the corresponding TaqMan probe IDs, reference sequences, exon boundaries, and amplicon lengths (in base pairs). The species designation “Oa” refer to Ovis aries, while “Cf” represents Canis familiaris (with 100% cross-reactivity). The putative reference genes include ACTB, HPRT1, and TBP. The target genes are NGF, NTRK1, p75NTR, TLR4, COX2, and STAR.

Gene Symbol	TaqMan ID	Sequence ID	Exons	Bp
NGF (nerve growth factor)	Cf02697134_s1	NM_001194950.1	1	161
NTRK1 (Neurotrophic Receptor Tyrosine Kinase 1)	Oa04767849_g1	XM_027976575.1	14–15	114
p75NTR (p75 neurotrophin receptor)	Oa04853013_m1	XM_027974687.1	2–3	62
TLR4 (Toll-Like Receptor 4)	Oa04656419_m1	NM_001135930.1	1–2	108
COX2 (cyclooxygenase 2)	Cf02625600_g1	NM_001003354	4–5	90
STAR (steroidogenic acute regulatory protein)	Oa04657047_m1	NM_001009243.1	4–5	69
ACTB (Beta-Actin)	Cf04931159_m1	NM_001195845.2	1	52
HPRT1 (Hypoxanthine Phosphoribosyltransferase 1)	Oa04825272_gH	XM_015105023.2	7–8	52
TBP (TATA Binding Protein)	Oa04818075_m1	XM_015097549.2	4–5	66

## Data Availability

The data supporting the findings of this study are included within the article. Further inquiries can be directed to the corresponding authors, Gabriella Guelfi (gabriella.guelfi@unipg.it) and Rolando Pasquariello (rolando.pasquariello@unimi.it).
